# Unbiased Analysis of Temporal Changes in Immune Serum Markers in Acute COVID-19 Infection With Emphasis on Organ Failure, Anti-Viral Treatment, and Demographic Characteristics

**DOI:** 10.3389/fimmu.2021.650465

**Published:** 2021-06-11

**Authors:** Krzysztof Laudanski, Hajj Jihane, Brook Antalosky, Danyal Ghani, Uyen Phan, Ruth Hernandez, Tony Okeke, Junnan Wu, Daniel Rader, Katalin Susztak

**Affiliations:** ^1^ Department of Anesthesiology and Critical Care, The University of Pennsylvania, Philadelphia, PA, United States; ^2^ Leonard Davis Institute for Healthcare Economics, The University of Pennsylvania, Philadelphia, PA, United States; ^3^ Department of Neurology, The University of Pennsylvania, Philadelphia, PA, United States; ^4^ School of Nursing, Widener University, Philadelphia, PA, United States; ^5^ College of Arts and Sciences, Drexel University, Philadelphia, PA, United States; ^6^ School of Biomedical Engineering, Drexel University, Philadelphia, PA, United States; ^7^ Department of Genetics, The University of Pennsylvania, Philadelphia, PA, United States; ^8^ Department of Nephrology, The University of Pennsylvania, Philadelphia, PA, United States

**Keywords:** COVID-19, remdesavir, convalescent plasma, hydroxychloroquine, steroids, MCP, CCL23, programmed death

## Abstract

Identification of novel immune biomarkers to gauge the underlying pathology and severity of COVID-19 has been difficult due to the lack of longitudinal studies. Here, we analyzed serum collected upon COVID-19 admission (t1), 48 hours (t2), and seven days later (t3) using Olink proteomics and correlated to clinical, demographics, and therapeutic data. Older age positively correlated with decorin, pleiotrophin, and TNFRS21 but inversely correlated with chemokine (both C-C and C-X-C type) ligands, monocyte attractant proteins (MCP) and TNFRS14. The burden of pre-existing conditions was positively correlated with MCP-4, CAIX, TWEAK, TNFRS12A, and PD-L2 levels. Individuals with COVID-19 demonstrated increased expression of several chemokines, most notably from the C-C and C-X-C family, as well as MCP-1 and MCP-3 early in the course of the disease. Similarly, deceased individuals had elevated MCP-1 and MCP-3 as well as Gal-9 serum levels. LAMP3, GZMB, and LAG3 at admission correlated with mortality. Only CX3CL13 and MCP-4 correlated positively with APACHE score and length of stay, while decorin, MUC-16 and TNFRSF21 with being admitted to the ICU. We also identified several organ-failure-specific immunological markers, including those for respiratory (IL-18, IL-15, Gal-9) or kidney failure (CD28, VEGF). Treatment with hydroxychloroquine, remdesivir, convalescent plasma, and steroids had a very limited effect on the serum variation of biomarkers. Our study identified several potential targets related to COVID-19 heterogeneity (MCP-1, MCP-3, MCP-4, TNFR superfamily members, and programmed death-ligand), suggesting a potential role of these molecules in the pathology of COVID-19.

## Background

Individuals infected with severe acute respiratory syndrome coronavirus 2 (SARS-Cov-2; COVID-19) can be asymptomatic or exhibit mild to morbid symptoms depending on viral load and individual characteristics (1–5). Blood pressure variation, impairments in oxygenation delivery, and metabolomics disruptions are examples of culprits seen in COVID-19 viral septicemia (3, 6, 7). The evolution and characteristics of immune system activation are critical for determining the overall clinical outcomes of SARS-Cov-2 infection and other critical care illnesses, as well as the emergence of failure of specific organs (7–16). For example, it has been observed that lung function deteriorates by the direct cytotoxic effect of the virus and local inflammation governed by monocyte, T cells, and neutrophils during viral sepsis, including SARS-Cov-2 (6, 17–19). In another example, kidney function impairment in COVID-19 is triggered by vascular dysfunction and monocyte activation, which seemed to have distinct immune characteristics as compared to those seen in other organ failures (12, 14, 15, 17). The emergence of the brain’s failure is linked to specific endothelial inflammation modulated by leukocytes (17, 20, 21). These dysfunctions may be related to the influx of highly activated monocyte and T-cells into any of the organs and potentiate pathological consequences for COVID-19. Alternatively, immune system markers signify organ failures without being a cause of dysfunction (21).

So far, most of the analysis of COVID-19 inflammation and end-organ failures had focused on global dysfunction, primarily when proteomics serum analysis was employed (22). Other analyses suffered from a small sample size (23). The large-scale proteomics analysis’s primary goal is to deliver clues to potential mechanisms leading to disease pathology, especially if combined with longitudinal analysis of marker dynamics (1, 11, 23, 24). However, such studies generate large amounts of data that are often difficult to put in the context of illness. Our study addresses this knowledge gap by focusing on selected monocyte and T cell activation biomarkers as suggested in editorial and reviews and some preliminary studies (4, 6, 12, 13, 25–27). Furthermore, the initial targets are infrequently examined for the causative role. Here, we applied a different approach than multi-omics analysis to be the hypothesize-driven targeted approach to a set of biomarkers.

The treatments in COVID-19 should modulate the direct viral cytotoxicity and immune system response to infection (7, 28, 29). The ideal therapy for viral septicemia should reverse immune dysfunctions, normalize immunological mediators, and reverse organ failures (7, 11, 24). However, it is unclear how any proposed treatments for COVID-19 would alter the immunological reaction in the yet-to-be-described immunological landscape of the COVID-19 disease. So far, most of the trials failed to deliver a cure (hydroxychloroquine, convalescent plasma) (30–32). Some have significant clinical gains (remdesivir) or were demonstrated to benefit a specific subsequent of patients (steroids) (32–36). Regardless of clinical efficacy, their effects on modulating immunological serum signatures remain mostly unknown. We hypothesize that by analyzing the effect of the COVID-19 treatment on the immune system activation profile, we will discern potential key mechanisms leading to recovery due to the implemented therapy.

In summary, we sought to analyze immunological profile markers related to monocyte and T cells activation during immune system activation using unbiased quantitative targeted proteomics during the evolution of COVID-19. We planned to relate observed changes to demographic variables, pre-existing comorbidities, clinical status, organ dysfunction, COVID-19 outcomes, and treatment. The goal was to identify potential targets linked to the emergence of COVID-19 organ dysfunctions for future research and therapy.

## Materials And Methods

### Study Population

Our study protocol was authorized by the Institutional Review Boards (IRBs) of the University of Pennsylvania and was performed according to the ethical guidelines of the 2003 Helsinki Declaration. Written informed consent was obtained from all enrolled patients.

The electronic medical records (EMR) EPIC was used to collect the demographic and medical data on all the enrolled participants. Patients self determined race and ethnicity. The Acute Physiology And Chronic Health Evaluation II (APACHE II) was calculated within one hour (APACHE_1hr_) and at 24 hours after admission (APACHE_24hrs_) (37). The burden of chronic disease was calculated as Charlson Comorbidity Index (CCI) (38). The severity of the illness was determined by Marshalls Organ Dysfunction Score (MODS) and Sequential Organ Failure Assessment (SOFA) (39). The survival was determined at 28 days.

Data regarding therapies with ventilatory support, renal replacement therapy (RRT), and extra-corporal membrane (ECMO) were retrieved. The status of organ failures was characterized using a framework from the GlueGrant and organ dysfunction scores (39–41). Central Nervous System failure (CNS_f_) was determined as an altered mental status secondary to acute cerebrovascular accident, seizure, or delirium lasting over 72 hours. Respiratory failure (R_f_) was defined as a condition that requires biphasic non-invasive ventilation, intubation, or ECMO engagement. Cardiovascular failure (CVS_f_) was determined among patients with shock requiring vasopressors. Liver failure (L_f_) was identified in patients with severely elevated alanine aminotransferase (ALT), aspartate aminotransferase (AST), total bilirubin, or ammonia levels. Renal failure (AKI_f_) was determined by serum creatinine from baseline. Hematological failure (B_f_) was determined when hemoglobin measured less than 9[g%], or increased blood products.

We investigated the engagement of hydroxychloroquine, remdesivir, convalescent plasma, and steroids. Except for the latter, the treatments were highly protocolized per hospital policy according to the FDA recommendations for the given treatment. The presence of steroid treatment was determined as the engagement of any intravenous or oral glucocorticoid steroid compounds to treat COVID-19 pneumonia per healthcare provider notes, according to COVID-19 specific hospital policy.

### Sample Collection and Processing

Blood was drawn at three different time intervals (t_baseline_; n=36), followed by 48hours (t_48hrs_; n=28) and seven days (t_7d_; n=17). Blood was collected in heparinized vacutainer tubes (BD, Franklin Lakes, NJ) and put on ice. Downstream processing of biological specimen was done following BSL-2 enhanced standard. Serum was separated by collecting the top layer after spinning the line at 1000x*g*, 10 minutes, 4°C within 3 hours from collection. Aliquot serum was stored at -80°C. The serum was inactivated by incubation of 100µl of serum with 5% Tween-20 (Bioworld, Baltimore, MD) for 20 minutes at room temperature. The serum was then either shipped for analysis or locally tested with enzyme-linked immunoassays (ELISA) of interest.

The commercial supplier employed O-link technology to assess the serum level of immunological protein. We selected the specific kit targeting biomarkers based on the preliminary data and literature review (**Supplemental Material 1**) (1, 7, 11, 14, 20, 42, 43). Inactivated serum samples (n=64) collected from patients with COVID-19 were analysed using an Olink panel (OLINK Bioscience, Uppsala, Sweden). The kit provides a microtiter plate for measuring 92 protein biomarkers, with data presented as Normalized Protein Expression (NPX) values plotted against protein concentration (in pg/mL) (44). The obtained results are presented as dimensionless values allowing for comparison of the measured protein across different variables (44). Olink technology does not allow for relating different cytokines to each other (44). IL-6 was measured using a multiplex kit (Theromofisher, Waltham, MA) on MagPix machine (Luminex; Austin, TX). CCL23 and MCP-1 were measured by ELISA (Biolegend, San Diego, CA)

### Statistics

Lavene’s test and distribution plots were used to test the normality of variables. Parametric variables will be expressed as mean ± SD and compared using *t*-Student. Multiple groups were compared using ANOVA with subsequent *t*-Student tests for intra-group comparisons. For non-parametric variables, median (M_e_) and interquartile ranges (IR) will be shown with U-Mann-Whitney statistics employed to compare such variables. Correlations were calculated as *r^2^*-Pearson momentum. The clustering center was calculated using the k-mean using R package using an unbiased approach to identify subgroups of the markers’ serum levels. A double-sided *p*-value less than 0.05 will be considered statistically significant for all tests, but we prioritized a more conservative value of 0.01 or less in most of the analyses. Regression analysis was done with stepwise forward methods. The receiver area operator was calculated using discharge home as a binary predictor. Statistical analyses were performed with R, Statistica 11.0 (StatSoft Inc., Tulsa, OK), and SPSS v26 (IBM, Endicott, NY).

## Results

### Characteristics of Studies Population

Subject demographics and clinical characteristics are shown in **Table 1**. The cohort represents subjects previously reported requiring hospitalization due to COVID-19.

**Table 1:** Demographic and clinical characteristics of the studied individuals.

**Age**	Age [X ± SD]		63.1 ± 19.28		
	Below 60 [%]		41.7%		
	Over 60 [%]		58.3%		
**Gender**	Male [%]		52.8%		
	Female [%]		47.2%		
	Not reported [%]		0.0%		
**Race**	Caucasian [%]		16.7% (50% Latino)		
	Black [%]		69.4%		
	Other/unknown/Asian [%]		13.9%		
**Pre-existing conditions**					
Charleston Comorbidity Index [X ± SD]			3.8 ± 2.99		
MI [%]			0.0%		
CHF [%]			16.7%		
PVD [%]			8.3%		
CVA/TIA [%]			13.9%		
Dementia [%]			8.3%		
COPD [%]			16.7%		
CTD [%]			2.8%		
Peptic ulcer disease [%]			5.6%		
Liver disease [%]			2.8%		
DM [%]			33.3%		
Hemiplegia [%]			8.3%		
CKD [%]			27.8%		
Solid tumor [%]			11.1%		
Leukemia [%]			0.0%		
Lymphoma [%]			0.0%		
AIDS [%]			0.0%		
Smoking		Smoker [%]	38.9%		
		Nonsmoker [%]	61.1%		
Vaper [%]			0.0%		
Mortality [%]			27.8%		
Length of Stay [X ± SD]			18.2 ± 20.02		
ICU [%]			58.3%		
intubated [%]			44.4%		
ECMO [%]			16.7%		
APACHE admission +1hr [X ± SD]			12.4 ± 9.11		
APACHE admission +24hrs [X ± SD]			12.8 ± 7.04		
**MOF**			**t1**	**t2**	**t3**
CNSf [%]			19.4%	14.3%	20.8%
CVf [%]			30.6%	22.9%	25.0%
Rf [%]			33.3%	40.0%	41.7%
AKIf [%]			22.2%	28.6%	25.0%
Lf [%]			66.7%	60.0%	58.3%
Bf [%]			58.3%	54.3%	66.7%

MI, myocardial infarction; CHF, congestive heart failure; PVD, peripheral artery disease; CVA, cerebrovascular accident; TIA, transient ischemic stroke; COPD, chronic obstructive pulmonary disease; CTD, connective tissue disease; DM, diabetes mellitus; CKD, chronic kidney disease; AIDS, acquired immunodeficiency syndrome; ICU, intensive care unit; ECMO, extracorporeal membrane oxygenation; APACHE, acute physiology and chronic health evaluation; CNSf, central nervous system failure; CVf, cardiovascular failure; Rf, respiratory failure; AKIf, renal failure; Lf, lung failure; Bf, blood failure.

### The Effect of Age, Gender, and Race on the Natural History of COVID-19 Immune System Activation

**Figure 1:**
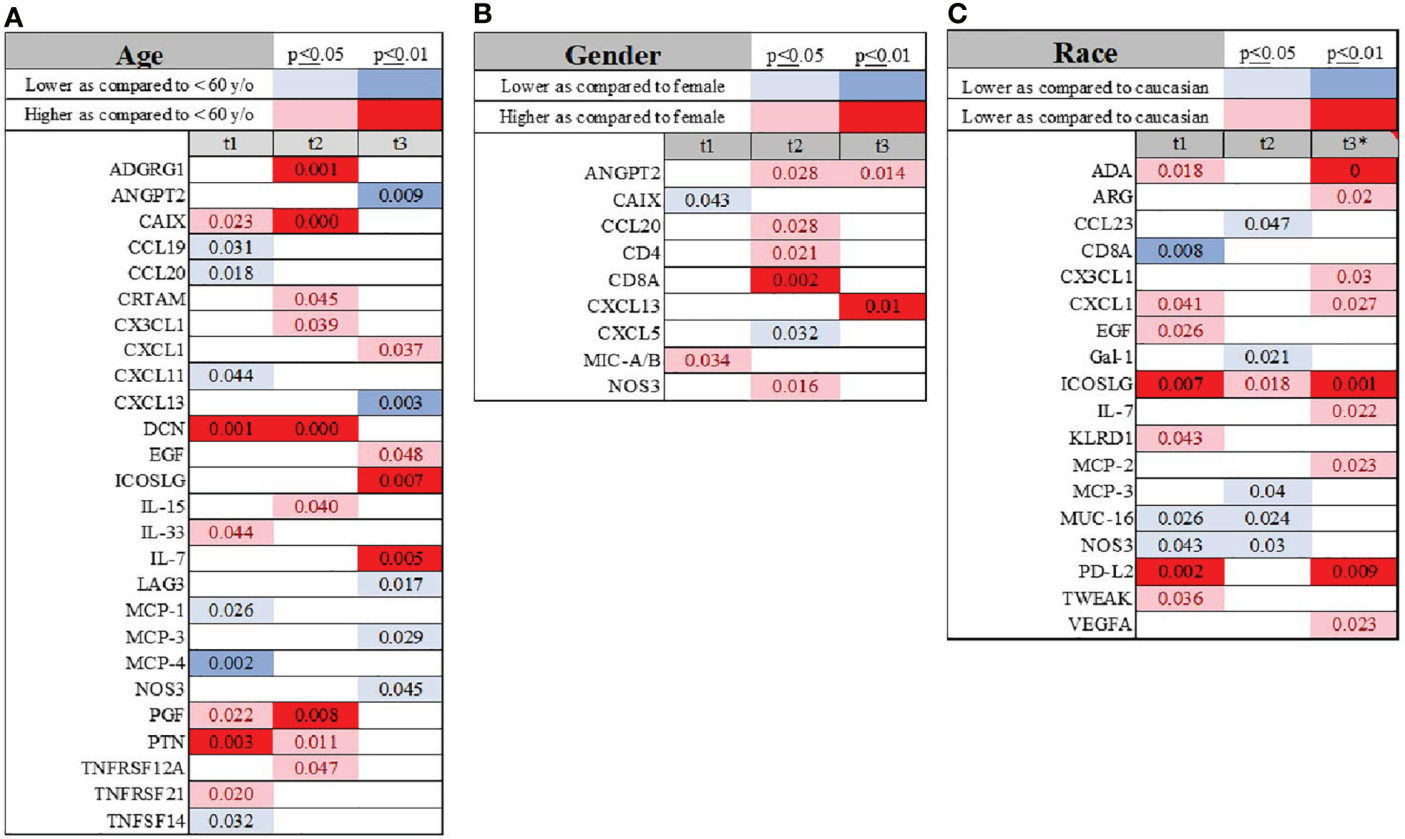
Visualization of temporal changes in the variation of the immunological biomarkers when age (cutoff of 60 years) **(A)**, gender **(B)** and race **(C)** were compared across the study subjects. Summary of statistically significant differences demonstrated as higher (red) or lower (blue) than an appropriate comparison group at standard [(p ≤ 0.05); pale shade] and more conservative (darker shade) The mean and SD of the compared group are listed in **Supplemental Material 2**. Denotation of abbreviations and corresponding UniProt number are listed in **Supplemental Material 2**.

Patients over the age of 60 showed lower levels of chemotaxis molecules (MCP-1, MCP-4, CCL20, CCL19) shortly after admission, while IL-33, CAIX, DCN, PGF, and PTN were higher (**Figure 1A** and **Supplemental Material 2**). After 7 days this pattern slightly changed. In addition, levels of ANGPT2, CXCL13, LAG3, and MCP-3 were lower, while IL-7, ICOS-L, EGF, and CXCL1 were higher as compared to younger subjects (**Figure 1A** and **Supplemental Material 2**). The correlation between serum level and age was highest for CAIX (t_admission_ *r^2^*[36]=0.448; p=0.006; t_+48hr_ *r^2^*[28]=0.557; p=0.001), DCN (t_admission_ *r^2^*[36]=0.548; p=0.001; t_+48hr_ *r^2^*[28]=0.789; p<0.000), ADGRG1 (t_admission_ *r^2^*[36]=0.399; p=0.016; t_+48hr_ *r^2^*[28]=0.55; p=0.002), CD28 (t_admission_ *r^2^*[36]=0.353; p=0.035; t_+48hr_ *r^2^*[28]=0.254; p=0.019), Gal-1 (t_admission_ *r^2^*[36]=0.427; p=0.009; t_+48hr_ *r^2^*[28]=0.311; p=0.107), PD-L1 (t_admission_ *r^2^*[36]=0.307; p=0.069; t_+48hr_ *r^2^*[28]=0.408; p=0.031), CX3CL1 (t_admission_ *r^2^*[36]=0.404; p=0.015; t_+48hr_ *r^2^*[28]=0.509; p=0.006), PTN (t_admission_ *r^2^*[36]=0.604; p=0.000; t_+48hr_ *r^2^*[28]=0.602; p=0.001; t_+7d_ *r^2^*[28]=0.503; p=0.040).

We did not observe a consistent pattern between males and females upon admission; however, after 48 hours, several molecules were higher in males compared to females, such as ANGPT2, CCL20, CD4, CD8, and NOS3 (**Figure 1B** and **Supplemental Material 2**).

Self-identified African-American individuals had lower serum levels of CD8a, MCP-3, CCL23, GAL, MUC-16, NOS3 and PD-L2, and higher ICOSLG levels (**Figure 1C** and **Supplemental Material 2**). 7 days into the disease’s progression, ADA, ICOSL, and PD-L2 were significantly elevated in African-American subjects, but CX3CL1, CXCL1, IL-7, and MCP-2 demonstrated somewhat less significant differences (**Figure 1C** and **Supplemental Material 2**).

### The Effect of Pre-Existing Comorbidities on the Serum Profile of the Immune System Activation

**Figure 2:**
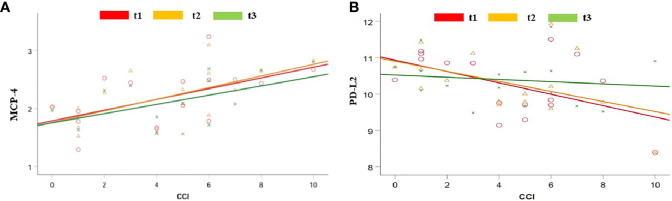
Regression analysis between pre-COVID-19 health conditions, calculated as the CCI and serum level of MCP-4 **(A)** and PD-L2 **(B)** when measured at admission (t1; red), 48 hours after admission (t2; yellow) and seven days after admission (t3; green).

The high burden of pre-COVID-19 comorbidities (CCI) correlated with MCP-4, PCN, DCN, CAIX, TWEAK, and TNFRS12A at admission and 48 hours afterward (**Figure 2A** and **Supplemental File 1**). PD-L2 was strongly linked to CCI at all three time points (**Figure 2B** and **Supplemental File 1**).

The presence of chronic kidney disease (CKD) was associated with two distinct marker patterns. The early pattern showed marked changes at admission and 48 hours later, as demonstrated by changes in IL-15, CD27, TNFRS9, PGF, CAIX, MCP-1, and MCP-4 (**Table 2**). In the delayed pattern at seven days after admission for COVID-19, significant differences were appreciated in serum levels of CD83, KRLD1, VEGFA, NCR, TNFSR24, PD-L2, and IL-12.

**Table 2:** The difference in serum immunological profile between patients with pre-existing CKD during duration of the COVID-19 infection.

Sample	No CKD/CKD	t1	t2	t3
IL-15	No CKD [X ± SD]	5.79 ± 0.556	**5.80 ± 0.680	*5.83 ± 0.834
	CKD [X ± SD]	5.88 ± 0.901	**6.82 ± 0.326	*6.64 ± 0.519
CD27	No CKD [X ± SD]	8.40 ± 0.497	*8.40 ± 0.508	**8.22 ± 0.375
	CKD [X ± SD]	8.72 ± 0.909	*9.08 ± 0.561	**9.09 ± 0.582
CAIX	No CKD [X ± SD]	*4.82 ± 0.918	**4.64 ± 0.830	**4.35 ± 0.722
	CKD [X ± SD]	*5.65 ± 1.101	**6.14 ± 0.981	**6.18 ± 1.024
MCP-1	No CKD [X ± SD]	11.43 ± 0.791	*11.61 ± 0.941	11.46 ± 1.012
	CKD [X ± SD]	11.42 ± 0.467	*12.66 ± 0.733	12.13 ± 0.800
MCP-4	No CKD [X ± SD]	**10.49 ± 0.819	**10.64 ± 0.795	10.44 ± 0.711
	CKD [X ± SD]	**9.55 ± 0.680	**9.62 ± 0.656	10.37 ± 0.579
TNFRSF9	No CKD [X ± SD]	*6.24 ± 0.648	**6.05 ± 0.807	5.88 ± 0.704
	CKD [X ± SD]	*6.92 ± 0.993	**7.20 ± 0.987	7.02 ± 1.160
PGF	No CKD [X ± SD]	7.42 ± 0.533	**7.33 ± 0.635	**7.17 ± 0.469
	CKD [X ± SD]	8.17 ± 1.225	**8.92 ± 0.863	**8.71 ± 0.800
CD83	No CKD [X ± SD]	3.09 ± 0.456	**3.02 ± 0.521	2.98 ± 0.442
	CKD [X ± SD]	3.47 ± 0.924	**4.05 ± 0.807	3.83 ± 0.908
KRLD1	No CKD [X ± SD]	**6.25 ± 0.545	*6.40 ± 0.564	6.39 ± 0.836
	CKD [X ± SD]	**7.28 ± 1.200	*7.09 ± 0.788	7.14 ± 0.882
VEGFA	No CKD [X ± SD]	*8.35 ± 0.557	**8.44 ± 0.740	**8.20 ± 0.399
	CKD [X ± SD]	*8.86 ± 0.470	**9.20 ± 0.395	**9.03 ± 0.408
NCR1	No CKD [X ± SD]	3.63 ± 0.668	*3.61 ± 0.692	3.45 ± 0.557
	CKD [X ± SD]	4.29 ± 1.211	*4.68 ± 1.112	4.31 ± 0.890
IL-12	No CKD [X ± SD]	5.61 ± 1.163	5.08 ± 1.250	4.86 ± 1.236
	CKD [X ± SD]	5.18 ± 1.231	5.55 ± 1.023	4.51 ± 0.917
PD-L2	No CKD [X ± SD]	2.10 ± 0.405	**2.10 ± 0.436	**1.91 ± 0.271
	CKD [X ± SD]	2.34 ± 0.523	**2.71 ± 0.218	**2.66 ± 0.117

*denote p value between CKN and non-CKD CKD at given time point between 0.05 and 0.01. **denote p value between CKD and non-CKD at given time point less than 0.01.

### Clinical Outcome and Serum Profile of Immune System Activation

**Figure 3:**
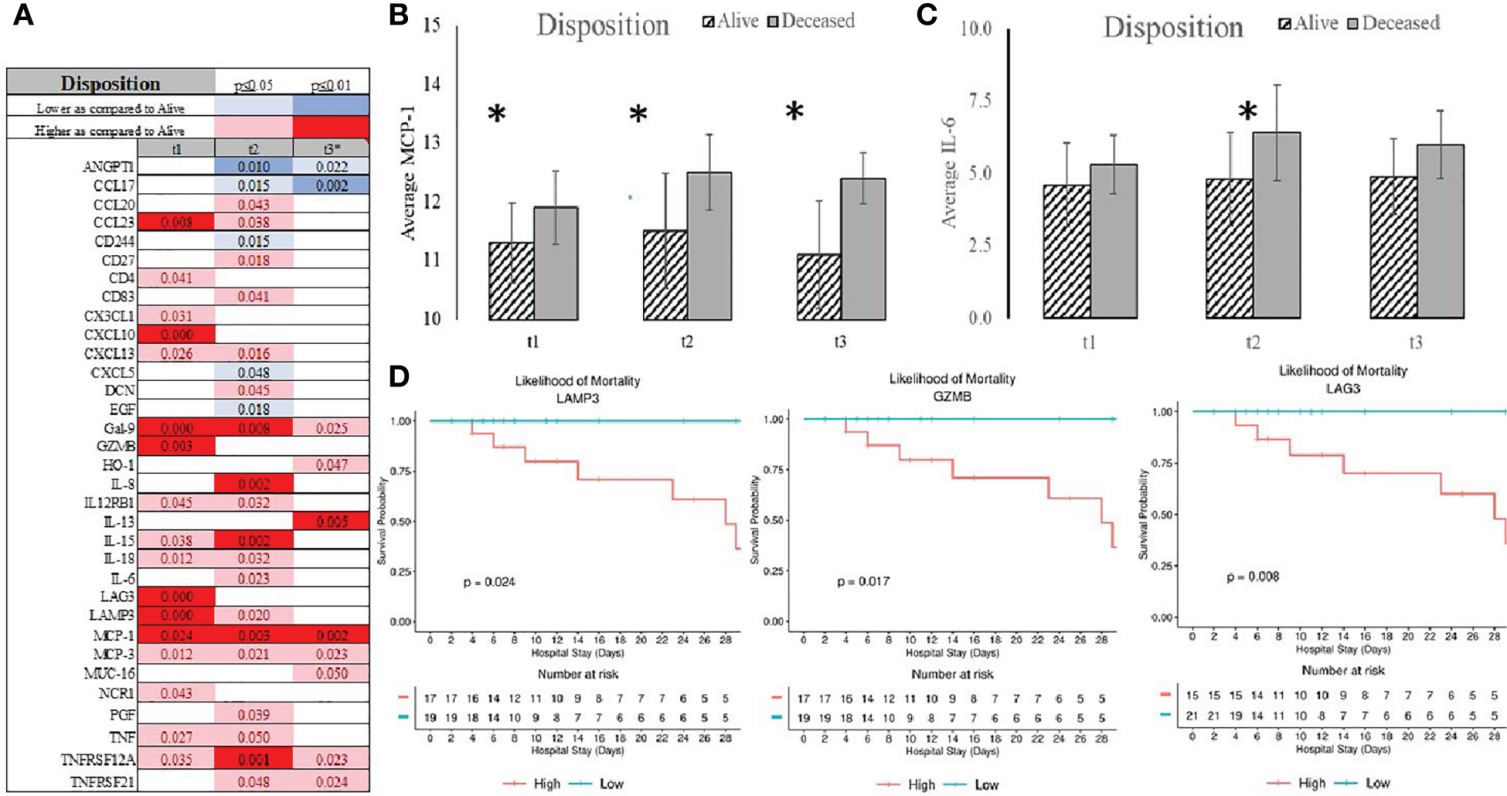
Summary of statistically significant differences demonstrated as higher (red) or lower (blue) than an appropriate comparison group at standard [(*p < 0.05); pale shade] and more conservative (darker shade) The mean and SD of the compared group are listed in **Supplemental Material 2**. Denotation of abbreviations and corresponding UniProt number are listed in **Supplemental Material 2**.

Non-survivors had higher serum levels of MCP-1, CCL23, CXCL10, GZ-MB, LAG3, and LAMP3 as compared to the survival group (**Figures 3A–C** and **Supplemental Material 2**). Gal-9, IL-8, MCP-1, and TNFRS12A were higher in the non-survivor group for 48 hours. It was noteworthy that MCP-1, MCP-3, and Gal-9 remained elevated in non-survivors at 7 days (**Figures 3A, B** and **Supplemental Material 2**). In contrast, ANGPT1 and CCL17 were markedly lower, and IL-6 was higher two and seven days after admission in non-survivors (**Figures 3A, C** and **Supplemental Material 2**). Analysis of the survival identified serum levels of MCP-1, GZMB, LAG3, and LAMP3 at admission were determinants of survival (**Figure 3D**). However, their ability to predictor mortality was relatively low satisfactory in terms of specificity and sensitivity (**Supplemental Figure 1A**). Serum level of EGF at admission demonstrated more satisfactory characteristics to distinguish patients with the ability to be discharged home (**Supplemental Figure 1B**).

**Figure 4:**
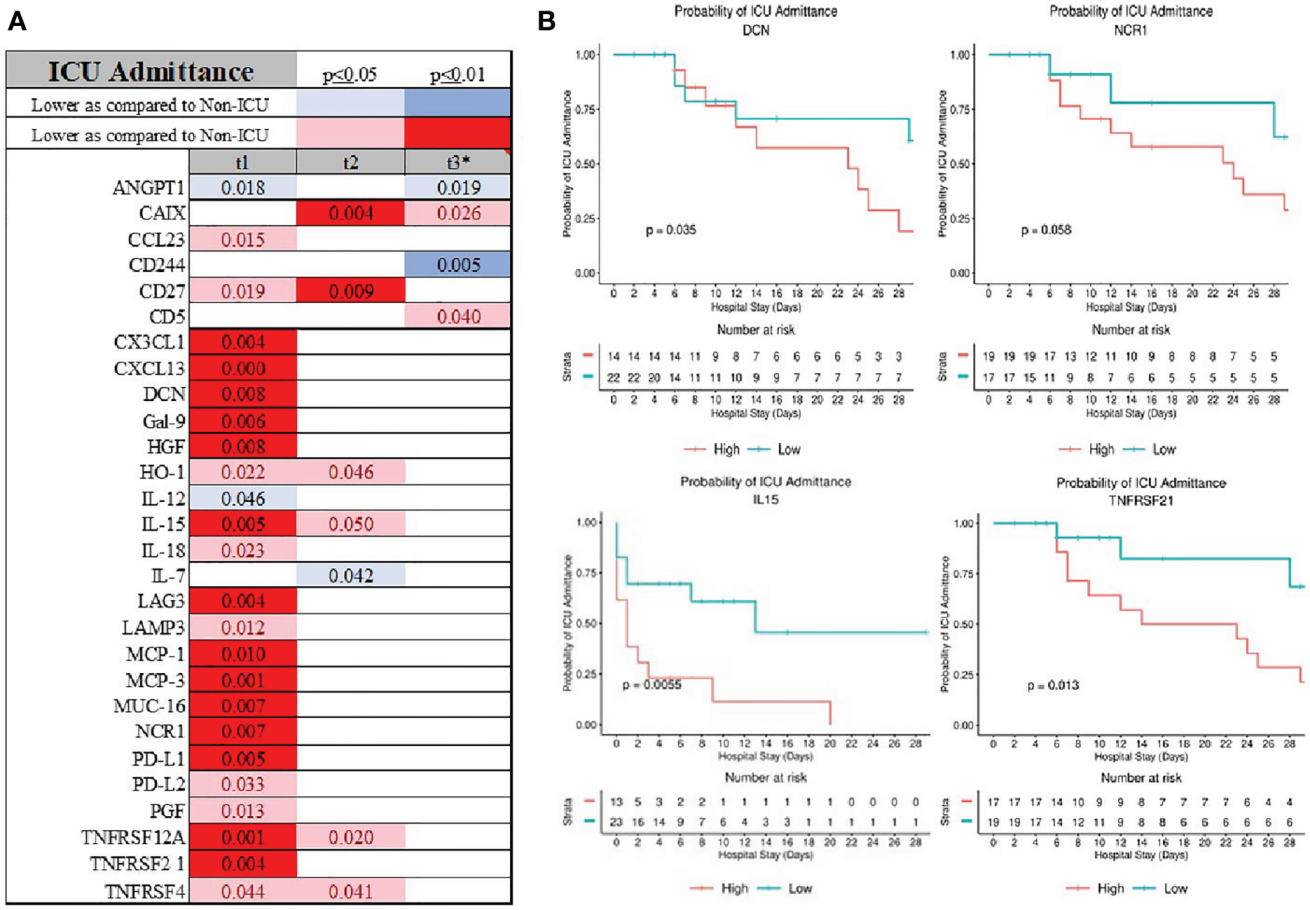
Also, patients who were admitted to the ICU had distinguish inflammatory profile at the admission **(A)** with DCN, NCR1, IL-15 and TNFRS21 being good predictor of ICU admission **(B)**.

Length of stay was best predicted be admission serum level of CCL23, Il-8, GZMB, CD244, GZMH, CCL4, and KIRLD3 (**Supplemental Figure 2**). Admission to the ICU was signified by elevation in several chemokines (CXCL1, CXCL13, MCP-1, MCP-3, CCL23), cytokines (IL-8, IL-15), immunological receptors (PD-L1, PD-L2, LAMP3, LAG3, CD27), TNFα receptor superfamily (TNFRSF12A, TNFRSF4), and stress response markers (HGF, NCR1, MUC-16) (**Figure 4A** and **Supplemental Material 2**). ANGPT1 and CD244 were consistently depressed in the serums of the patients admitted to the ICU. Admission serum level of decorin (DCN), natural cytotoxicity triggering receptor 1 (NCR1), CD28, and TNFRSF21 were determinants of admission to the ICU (**Figure 4B**).

**Figure 5:**
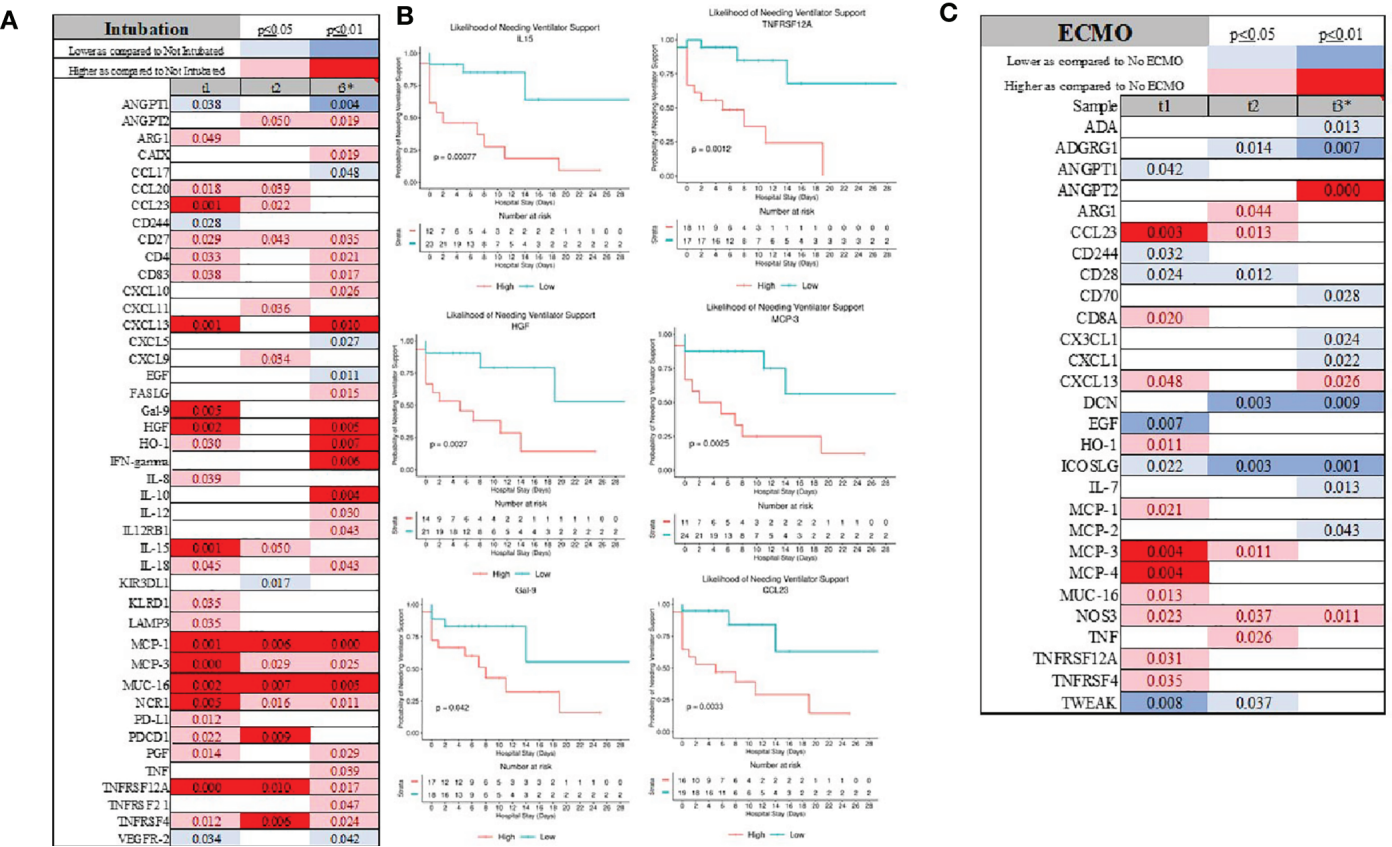
Intubation was signified for several markers **(A)** which also discriminate patient at the admission for those requiring ventilatory support **(B)**. The profile of patient requiring ECMO was significantly different considering large number of inflammatory marker being depressed as compared to patient not requiring ECMO **(C)**.

A similar serum immunological pattern was seen in patients needing intubation and ventilatory support. Chemokines (CXCL13, MCP-1, MCP-3, CCL20, CCL23), cytokine (IL-15), immunological receptors (CD4, CD27, CD83, LAMP3, PD-L1, PD-L2, PDCD1), TNFα receptor superfamily members (TNFRSF12A, TNFRSF4), and HGF were elevated in patients needing intubation (**Figure 5A** and **Supplemental Material 2**). Concomitantly, ANGPT1, CCL17, CD244, and EGF were lower, but only ANGPT1 had consistently and statistically significantly depressed serum levels (**Figure 5A** and **Supplemental Material 2**). TNFα, IL-10, IL-12, IL-18, and IFNγ were higher - but only seven days into ICU admission as compared to the non-intubated patients (**Figure 5A** and **Supplemental Material 2**). Finally, initial serum levels of IL-15, HGF, MCP-3, Gal-9, CCL23, and TNFRSF12A were distinguished patients needing ventilatory support with more favorable outcomes (**Figure 5B**).

Patients who required ECMO showed distinct immunological patterns as signified by lower serum levels of CD28, CD224, and ICOS-L as compared to the non-ECMO group (**Figure 5C** and **Supplemental Material 2**). ANGPT1 was also detectable at a lower level, a similar pattern as that shown in the patients requiring intubation (Supplemental Material 2). As compared to an intubated group, CXCL13, MCP-1, MCP-3, and CCL23 were also elevated. The most striking discovery in the ECMO group was the decrease in several markers at seven days (t3) after admission to the hospital, including ADA, ADGRG1, CD70, CX3CL1, CXCL1, IL-7, and MCP-2, while ANGPT2 was significantly elevated (**Figure 5C** and **Supplemental Material 2**).

**Figure 6:**
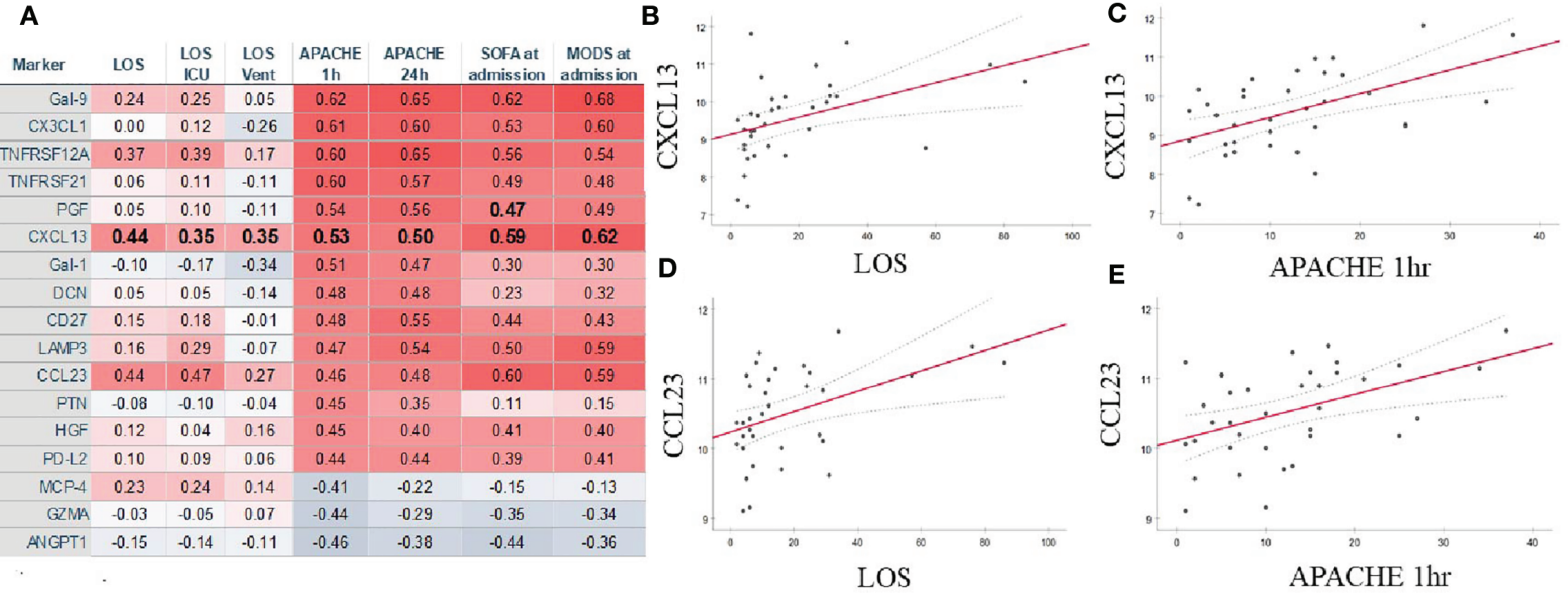
Several markers correlated strongly with numerous measures of COVID-19 severity **(A)**. CXCL-13 **(B, C)** and CCL23 **(D, E)** demonstrated strong correlations with admission APACHE_1hr_ **(B, D)** and the length of stay in the hospital **(C, D)**. Summary of statistically significant differences demonstrated as higher (red) or lower (blue) correlation. LOS, length of stay; ICU, intensive care unit; Vent, mechanical ventilation; APACHE, acute physiology and chronic health evaluation; SOFA, sequential organ failure assessment; MODS, multiple organ dysfunction syndrome.

Admission APACHE score correlated strongly with serum Gal-9, CX1CL3, TNFRS12A, TNFRS21, PGF, CXCL13, Gal-1, DCN, CD27, and CCL23 levels. ANGPT, GZMA, and MCP-4 exhibited strong negative correlations with the illness severity index at admission (**Figure 6A** and **Supplemental Pivot Table 1**). Length of stay, APACHE, SOFA, and MODS was associated with serum level of CXCL13 and CCL23 (**Figures 6A–E** and **Supplemental Pivot Table 1**). MCP-4 correlated positively with LOS but negatively with APACHE (**Figures 6A, D**). Admission serum IL-6 level correlated with overall length of stay (*r^2^*[36]=0.36; p=0.037), but not with admission APACHE (*r^2^*[36]=-0.12; *p*=0.487), MODS (*r^2^*[36]=0.145; *p*=0.398), or SOFA (*r^2^*[36]=0.102; *p*=0.553) (**Supplemental Pivot Table 1**).

### End Organ Failure and Serum Patterns of Organ Failures

**Figure 7:**
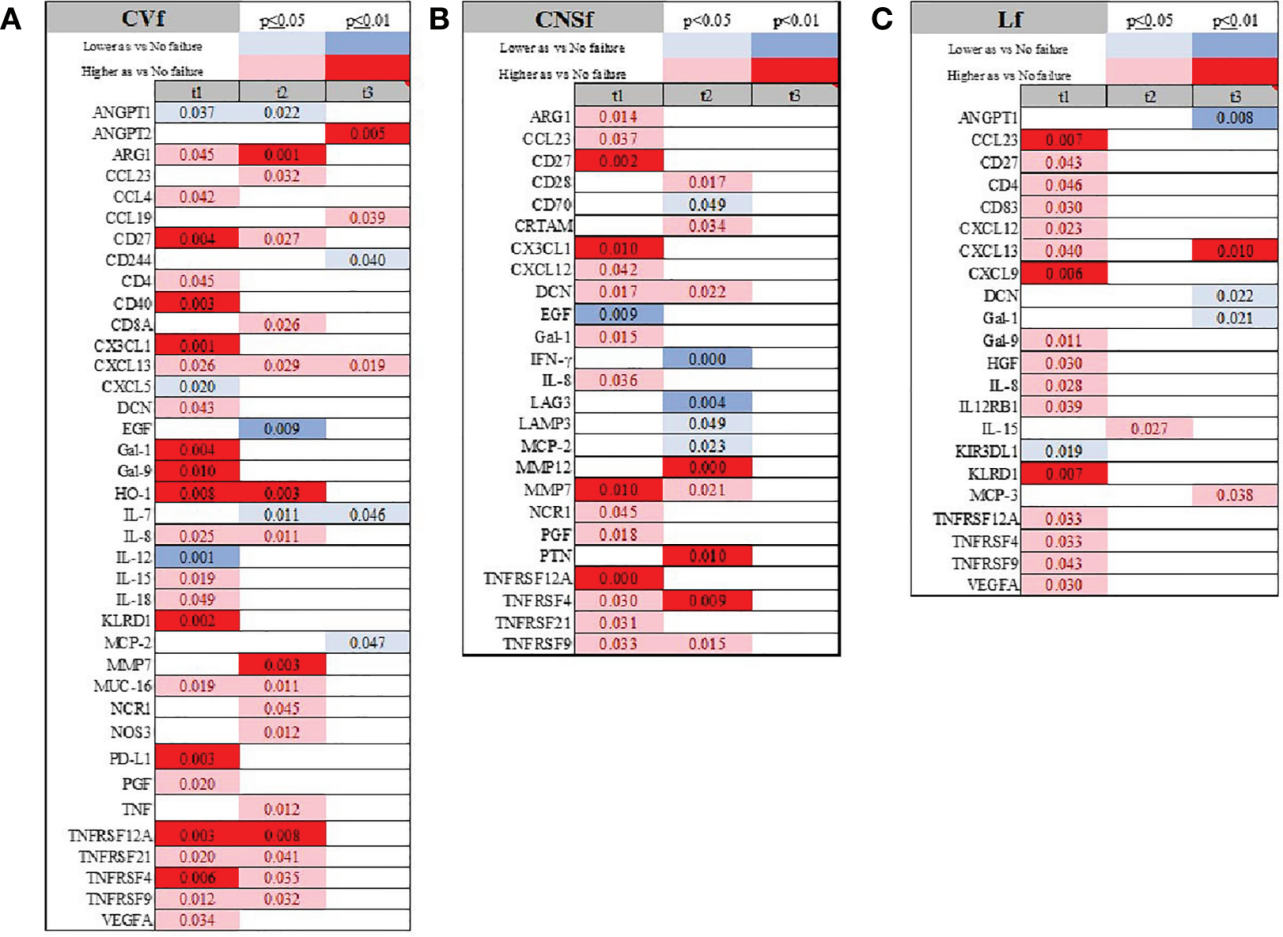
Time-related temporal markers of the immune system for cardiovascular failure (CVf; A), central nervous system failure (CNSf; B), and liver failure (Lf; C). Summary of statistically significant differences demonstrated as higher (red) or lower (blue) than an appropriate comparison group at standard [(p ≤ 0.05); pale shade] and more conservative (darker shade) The mean and SD of the compared group are listed in **Supplemental Material 2**. Denotation of abbreviations and corresponding UniProt number are listed in **Supplemental Material 2**.

Patients with cardiovascular failure and hypotension requiring intravenous pressors showed lower IL-7 levels later in their COVID-19 trajectory, while CXCL5, IL-12, and ANGPT-1 levels were depressed early during the course of infection (**Figure 7A**; **Supplemental Figure 1A** and **Supplemental Material 2**). CD40, CXCL5 and IL-12 were the lone markers exclusively altered at admission if a patient presented with CVf only. Patients presented with central nervous system failure showed increased ARG-1, CX3CL1, and DCN, but lower serum expression of EGF at admission (**Figure 7B**; **Supplemental Figure 1** and **Supplemental Material 2**). The most striking feature of liver failure was the elevation of CCL23, FGF2, and KRLD1 and depression of KIR3DL1 at admission, while Gal-1 and DCN were decreased later during the history of COVID-19 (**Figure 7C** and **Supplemental Material 2**). KIR3DL1 and CXCL9 were unique markers for liver failure (**Supplemental Figure 1B**). Anemia was not related to specific immune system activation patterns in serum (data not shown).

**Figure 8:**
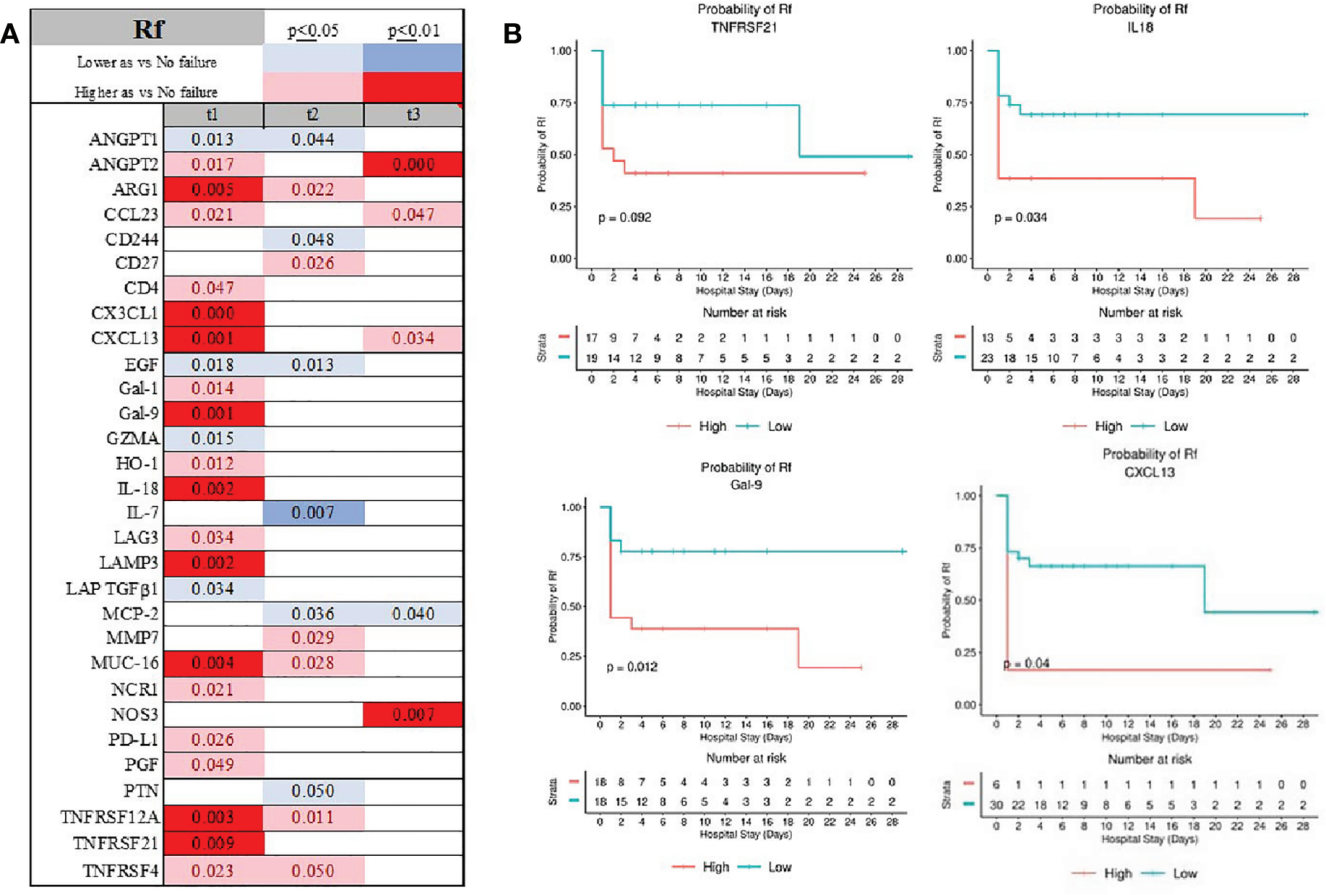
Time-related temporal markers of the immune system for cardiovascular failure (Rf) **(A)** demonstrated IL-18, GAL-9, CXCL13 and TNFRS2 being predictors of patients requiring ventilatory support **(B)**. Summary of statistically significant differences demonstrated as higher (red) or lower (blue) than an appropriate comparison group at standard [(p ≤ 0.05); pale shade] and more conservative (darker shade) The mean and SD of the compared group are listed in **Supplemental Material 2**. Denotation of abbreviations and corresponding UniProt number are listed in **Supplemental Material 2**.

Respiratory failure was signified by depression of EGF, GZMA, LAP, IL-4, and IL-7, and increased expression of MUC-16, ARG-1, and LAMP-3 (**Figure 8A** and **Supplemental Figure 1** and **Supplemental Material 2**). Depression in GRZMA was unique for Rf at admission (**Supplemental Figure 1B**). Finally, admission serum TNFRSF21, IL-18, Gal-9, and CXCL13 were associated with a respiratory decline during hospital stay (**Figure 8B**).

**Figure 9:**
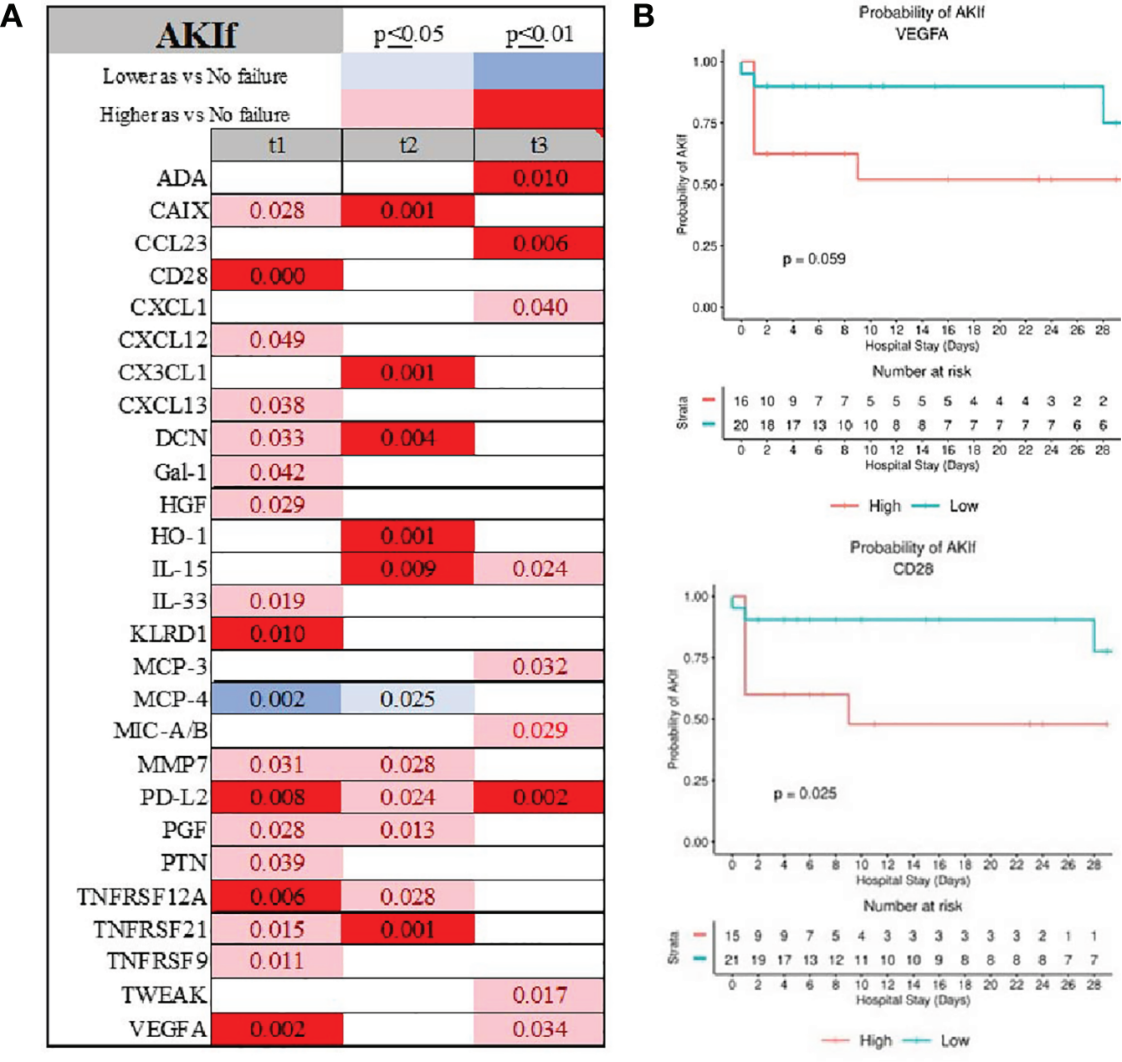
Time-related temporal markers of the immune system for cardiovascular failure (AKIf) **(A)** demonstrated VEGFA and CD28 being predictors of patients experiencing acute kidney failure during hospitalization **(B)**.Summary of statistically significant differences demonstrated as higher (red) or lower (blue) than an appropriate comparison group at standard [(p ≤ 0.05); pale shade] and more conservative (darker shade) The mean and SD of the compared group are listed in **Supplemental Material 2**. Denotation of abbreviations and corresponding UniProt number are listed in **Supplemental Material 2**.

The emergence of AKI was demonstrated by elevated expression of VEGFA, CD28, TNFRSF21, and KRLD1, while MCP-4 was consistently depressed (**Figure 9A**; **Supplemental Figure 1** and **Supplemental Material 2**). CAIX, IL-33, MCP, and PD-L2 were uniquely abnormal in patients with AKIf at admission (**Supplemental Figure 1B**). Initial serum level of VEGFA and CD28 determined the emergence of AKI during admission (**Figure 9B**).

We identified several proteins whose levels showed highly dynamic changes during COVID-19 infection (**Supplemental Figures 1A, C**). Elevated levels of CXCL13, MMP-7, Gal-1, PDF, and TNFRS family (TNFRSF21, 12A, 4, and 9) during the first 48 hours after admission to the hospital were present across several failures (**Supplemental Figure 3C**).

### The Effect of the Treatment on the Immunological Profiling

**Figure 10:**
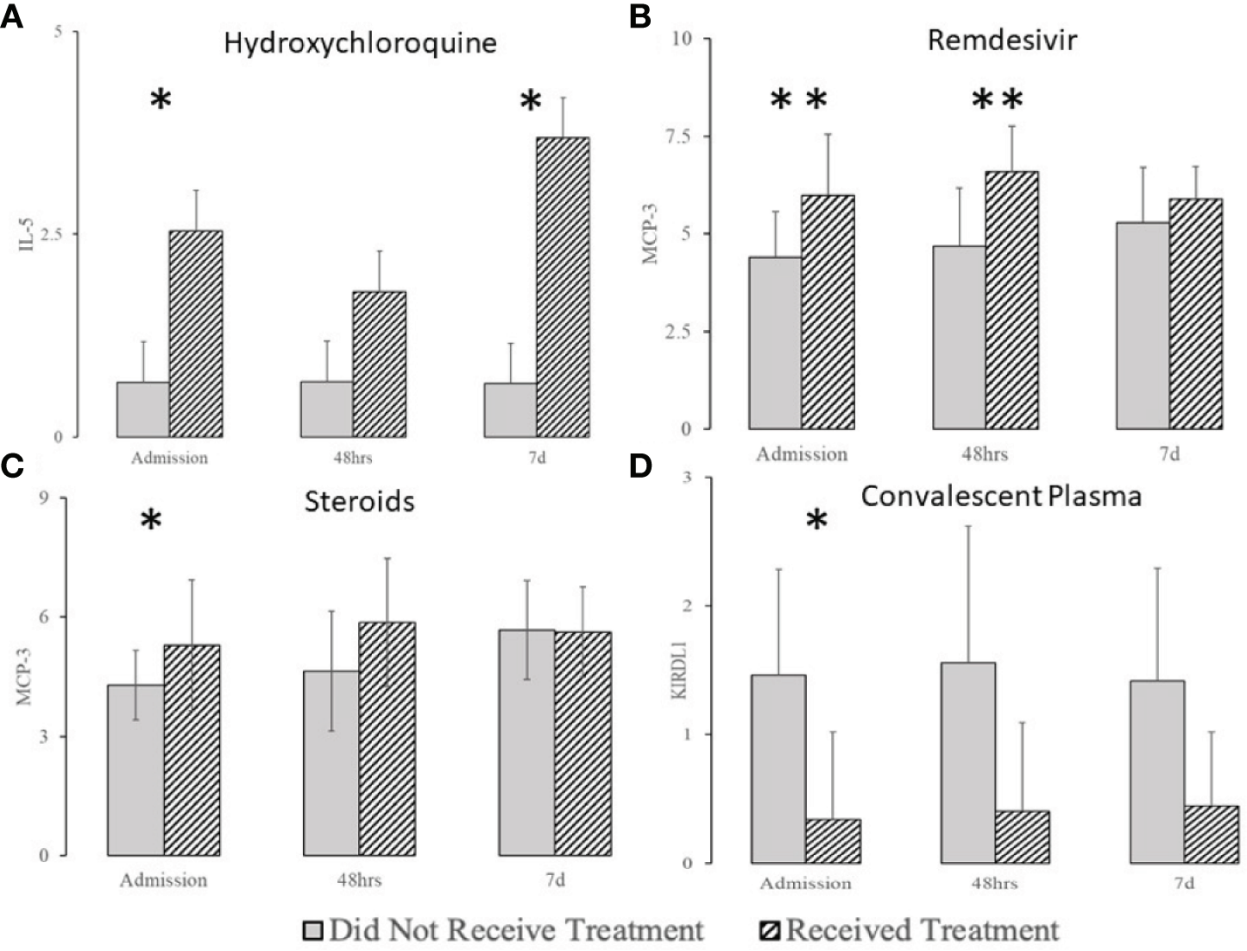
The effect of hydroxychloroquine **(A)**, remdesivir **(B)**, steroids **(C)**, and convalescent plasma **(D)** on selected biomarkers (complete list in **Supplemental Figure 2**). *denotes the difference p ≤ 0.05, **denotes the difference p ≤ 0.01.

Hydroxychloroquine had an almost negligible effect on most of the markers except for IL-5 (**Figure 10A**). Remdesivir treatment interacted with serum levels of ARG-1, CD28, IL-4, and MCP-1, and MCP3 early at presentation (**Figure 10B** and **Supplemental Figure 4**). Steroids affected Gal-9, IL-12, and MCP-3 early and affected EGF, NOS3, and VEGFR3 levels later in the COVID-19 disease course (**Figure 10C** and **Supplemental Figure 4**). Treatment with convalescent serum interacted with CXCL10, CXCL13, IL-12RB1 and KIR3DL1 (**Figure 10D** and **Supplemental Figure 4**).

## Discussion

Immunological serum profiling revealed that certain biomarkers are virtually uniformly linked to less favorable outcomes (TNFRS12A, TNFRS9, TNFSR21, Gal-1, CD27, MCP family, and CCL23), while others may be related to specific organ injury patterns (CCL4, CD40, CXCL5, and IL-15 for cardiovascular failure; IL-12RB1, KIR3DL1, and CXCL9 for liver failure; CAIX, IL-33, and CXCL9 for kidney failure; GZMA for respiratory failure). EGF was the only predictor of discharge home with somewhat satisfactory operator characteristics. We also noticed remarkable dynamism of the markers throughout the disease. Gender and race differences between different serum markers were present but were of unclear pathophysiological significance. Finally, we demonstrated the limited effects of the medical therapies on the serum immunological profile.

The predominant activation of several tumor necrosis family members, IL-15, CCL23, NCR1, and CD27 suggests extensive activation of the T cells as well as NK cell (45–47). IL-15, NCR1, and CCL23 are critical for the proliferation of the NK and T cells (25, 45, 48–50). The increased number of the T and NK cells is mainly supported by IL-15 that is critical for preventing activation-induced apoptosis (48, 49). Elevated expression of CD27 significantly augments these processes, as it supports proliferation and inhibits apoptosis (51). Concomitant elevation in TNFRS2 and TNFRS4 further suggests sustained and prolonged T cells’ activation (52–55). The net effect is an increase in cell activation and activity, including granzyme release, as demonstrated in our dataset. The results may suggest a surge in the T and NK activity, a frequent observation in COVID patients, and other viral infections (17, 19, 23, 28, 45).

The increase in IL-15 secretion is linked to lung injury in influenza by promoting the influx of CD8^+^ T cells (19, 42). Both IL-18 and IL-15 were reported as potential factors contributing to respiratory demise in COVID-19 patients (1, 22, 27, 56). CD27 and increased expression of CXCL13 may support blast response from B cells, as is seen in COVID-19, further propelling vascular inflammation and lung injury (57). This inflammatory process is further fueled by significant activation of the VEGF activation pathways, as seen in our dataset (58). Together with CD27 and TWEAK activation, this is highly suggestive of ongoing vasculitis, a common feature of organ dysfunction in COVID-19 (51, 59, 60). The observed pattern of immunological markers resembles the response to viremia in general (19, 47). This profile is highly supportive for recruiting T and NK cells and, to a lesser extend, other leukocytes (MO, neutrophils) into the lung and kidney during inflammation (61, 62). It likely that recruitment may underlie the inflammatory process damaging the end organs in COVID-19 as it is seen in other viral infections where self-sustained autoinflammatory processes emerge (19, 48, 55). The emergence of self-sustained activation of T and NK cells may suggest why COVId-19 extands beyond the initial insult.

We found that process of immune activation is relatively selective. An analysis of cytokine patterns found elevated IL-6 levels, but other cytokines implicated in mediating cytokine storm were not similarly affected. Instead, the immunological activation profile is highly suggestive of the T and NK cell activation instead of widespread activation as often suggested in “cytokine storm” (9, 11, 63). This activation of T cells and NK cells Elevation in Fas and some of the TNSFR family implicates high apoptosis activity, suggesting high cell turnover, a typical feature of sepsis (11, 60).

We described several potential organ-specific immune system activation patterns, but their effect on organ failure emergence is unclear (46, 64, 65). The only advent of kidney failure, liver failure, and need for pressors was associated with elevation of specific markers (CCL4, CD40, CXCL5, and IL-15), but our findings’ interpretation needs more broad context (66). Elevation in VEGFA and CD28 in patients with kidney failure suggests its involvement in the emergence of this organ failure (58, 67). Similarly to the lungs, the observed inflammatory profile suggests an environment where T cells and NK are activated. Their migration into lung or kidney tissue may be responsible for the induction of organ dysfunction (68). Our study suggests potential investigative targets, but the question of their causation in organ failure during COVID-19 is yet to be established.

The effects of treatments were variable and rarely reflected in changes in immunological patterns. Hydroxychloroquine has virtually no impact on immunological serum profile, consistent with its mechanism of action and lack of effectiveness in treating acute COVID-19 (69). Convalescent plasma affected immunological serum profile somewhat, finding coherent evidence of minimal effectiveness (33). Remdesivir suppressed activation of ARG1, soluble CD28, IL-4, IL-7, MCP-1, and MCP-3. These effects are probably secondary to suppressing viral proliferation (70). The effect of steroids was focused on altering IL-12 and MCP-3, yet its linkage to recovery from COVID-19 needs to be established. These results may demonstrate why some of these therapies have limited clinical impact.

The study has several limitations. Perhaps, the most significant one is the exploratory nature of the study. Though we targeted specific immunological markers based on a literature review, our study suggests a potential target without establishing if the marker is the culprit of the COVID-19-related demise or underlying cause (1, 7, 11, 14, 20, 42, 43). Olink technology limits cross-molecule comparisons (44). Hence, our study had to be limited to temporal changes in one marker level without commenting on composition as the difference in concentrations between different immunological modulators (44). Despite this limitation, Olink testing’s robustness was demonstrated by measuring the serum concentration of IL-6 and MCP-1, demonstrating elevation of both cytokines in patients with unfavorable outcomes (22, 27, 40).

The definition of organ failure used a well-established framework, but the data interpretation may be biased secondary to extraction from electronic medical records (40). Our definitions of organ failure are much more encompassing as compared to more restrictive studies (11, 63). Our study involved a heterogeneous population of patients. This allowed testing several hypotheses across several COVID-19 presentations in the context of comorbidities and demographical variables, yet hampered analysis of these variables on immunological profile, race, and clinical outcomes (28, 34, 41, 71). Race and sex were determined by patients, which is a subject of controversy.

Our study demonstrated significant effects of age and pre-existing renal failure on the immunological profile (28, 34, 41, 71). A more extensive study would be instrumental in accounting for these variables to analyze immunological serum profiles in COVID-19 patients. Some of these variables, like age, have an independent influence on the progression of pneumonia in general, while others (like a renal disease) affect immunological responses (14, 15, 71). Serum measurements may not reflect the molecules’ functional activity since we detected shed, released, or secreted molecules (44). These processes can be part of the immune response, transcyclation of a receptor, or molecule release secondary to cytolysis, necrosis, or apoptosis (48). The study is observational, and the result should be considered as hypothesis-generating. Despite that using longitudinal analysis demonstrated high longitudinal variability of the immune markers, the causative effect of any of these changes cannot be determined. A similar problem was seen in prior studies trying to pinpoint markers causative impact in other viral diseases (7, 28, 43). However, several chemokines were reported elevated in COVID-19 and other inflammatory conditions, suggesting, at minimum, potential investigational targets (9, 40, 43, 63). We also took a very conservative approach to analyze our data, frequently calling on statistical significance when the false-positive probability was less than 0.01 instead of the traditional 0.05 value as a cutoff for rejecting the null hypothesis. Finally, we applied multiple statistical methods to more in-depth described the immunological situation of patients.

Our study strongly suggests vibrant T & NK cell response during COVID-19 in our population (7–9, 11). Abnormalities in IL-15, NCR1, CD27, and an increase in T & NK cell activation traits, similar to those observed during swine flu and influenza, were prominent and may be considered a target of further investigation using a more controlled methodology (28). IL-15 was previously suggested to be a potential target in the treatment of COVID-19 viremia (29). When examining the MCP family’s role, several chemokines seemed also to be prominent in patients’ immunological signatures suggesting activation and trafficking of the MO. To determine causative effects of these molecules in the emergence of the COVID-19 morbidities will require *in vivo* experiments but our study identified several promising targets.

## Data Availability Statement

The datasets presented in this study can be found in online repositories. The names of the repository/repositories and accession number(s) can be found in the article/**Supplementary Material**.

## Ethics Statement

The studies involving human participants were reviewed and approved by Institutional Review Board at the University of Pennsylvania. The patients/participants provided their written informed consent to participate in this study.

## Author Contributions

KL: study concept, patient recruitment, sample processing, immunological measurements, data analysis, manuscript writing, manuscript review, submission. BA: data analysis, manuscript writing.

DG: data analysis, manuscript writing. RH: data analysis, manuscript writing. HJ: patient consent, sample acquisition clinical data collection. UP: clinical data collections, data analysis, manuscript preparation. TO: data analysis, manuscript preparation. DR: data analysis, manuscript preparation. KS: immunological profile measurements, data analysis, manuscript preparation. All authors contributed to the article and approved the submitted version.

## Funding

K23 GM120630, R01 DK105821, R01 DK087635, R01 DK076077.

## Conflict of Interest

The authors declare that the research was conducted in the absence of any commercial or financial relationships that could be construed as a potential conflict of interest.

## Supplementary Material

Supplementary Table 1 Pivot table of correlations between biomarkers and clinical indicators.
