# Supplementary material for: Unbiased Analysis of Temporal Changes in Immune Serum Markers in Acute COVID-19 Infection With Emphasis on Organ Failure, Anti-Viral Treatment, and Demographic Characteristics
Source: Front Immunol. 2021 Jun 11;12:650465. doi: 10.3389/fimmu.2021.650465 (PMC8226183; doi:10.3389/fimmu.2021.650465)

Supplementary Material

# **Supplemental Material #1**

The lineup of the multiplex kits used in the study, together with marker abbreviations and UniProt numbers.

| **Marker (abbreviation)** | **UniProt number** |
| --- | --- |
| Adenosine Deaminase (ADA) | P00813 |
| Adhesion G-protein coupled receptor G1 (ADGRG1) | Q9Y653 |
| Angiopoietin-1 (ANG-1) | Q15389 |
| Angiopoietin-1 receptor (TIE2) | Q02763 |
| Angiopoietin-2 (ANGPT2) | O15123 |
| Arginase-1 (ARG1) | P05089 |
| C-C motif chemokine 17 (CCL17) | Q92583 |
| C-C motif chemokine 19 (CCL19) | Q99731 |
| C-C motif chemokine 20 (CCL20) | P78556 |
| C-C motif chemokine 23 (CCL23) | P55773 |
| C-C motif chemokine 3 (CCL3) | P10147 |
| C-C motif chemokine 4 (CCL4 ) | P13236 |
| C-X-C motif chemokine 1 (CXCL1) | P09341 |
| C-X-C motif chemokine 10 (CXCL10 ) | P02778 |
| C-X-C motif chemokine 11 (CXCL11) | O14625 |
| C-X-C motif chemokine 13 (CXCL13 ) | O43927 |
| C-X-C motif chemokine 5 (CXCL5 ) | P42830 |
| C-X-C motif chemokine 9 (CXCL9 ) | Q07325 |
| Carbonic anhydrase IX (CAIX) | Q16790 |
| Caspase-8 (CASP-8 ) | Q14790 |
| CD27 antigen (CD27) | P26842 |
| CD40 ligand (CD40-L) | P29965 |
| CD40L receptor (CD40) | P25942 |
| CD70 antigen (CD70) | P32970 |
| CD83 antigen (CD83) | Q01151 |
| Cytotoxic and regulatory T-cell molecule (CRTAM) | O95727 |
| Decorin (DCN) | P07585 |
| Fas antigen ligand (FasL) | P48023 |
| Fibroblast growth factor 2 (FGF2) | P09038 |
| Fractalkine (CX3CL1 ) | P78423 |
| Galectin-1 (Gal-1) | P09382 |
| Galectin-9 (Gal-9) | O00182 |
| Granzyme A (GZMA) | P12544 |
| Granzyme B (GZMB) | P10144 |
| Granzyme H (GZMH) | P20718 |
| Heme oxygenase 1 (HO-1) | P09601 |
| Hepatocyte growth factor (HGF) | P14210 |
| ICOS ligand (ICOSLG) | O75144 |
| Interferon beta (IFN-beta) | P01574 |
| Interferon gamma (IFN-gamma) | P01579 |
| Interleukin-1 alpha (IL-1 alpha) | P01583 |
| Interleukin-10 (IL-10) | P22301 |
| Interleukin-12 (IL-12) | P29460, P29459 |
| Interleukin-12 receptor subunit beta-1 (IL12RB1) | P42701 |
| Interleukin-13 (IL-13) | P35225 |
| Interleukin-18 (IL-18) | Q14116 |
| Interleukin-2 (IL-2) | P60568 |
| Interleukin-21 (IL-21) | Q9HBE4 |
| Interleukin-33 (IL-33) | O95760 |
| Interleukin-35 (IL-35) | Q14213,P29459 |
| Interleukin-4 (IL-4) | P05112 |
| Interleukin-5 (IL-5) | P05113 |
| Interleukin-6 (IL-6) | P05231 |
| Interleukin-7 (IL-7) | P13232 |
| Interleukin-8 (IL-8) | P10145 |
| Latency-associated peptide transforming growth factor beta-1 (LAP TGF-beta-1) | P01137 |
| Lysosome-associated membrane glycoprotein 3 (LAMP3) | Q9UQV4 |
| Macrophage colony-stimulating factor 1 (CSF-1) | P09603 |
| Matrix metalloproteinase-12 (MMP-12) | P39900 |
| Matrix metalloproteinase-7 (MMP-7) | P09237 |
| MHC class I polypeptide-related sequence A/B (MIC-A/B) | Q29983,Q29980 |
| Monocyte chemotactic protein 1 (MCP-1) | P13500 |
| Monocyte chemotactic protein 2 (MCP-2) | P80075 |
| Monocyte chemotactic protein 3 (MCP-3) | P80098 |
| Monocyte chemotactic protein 4 (MCP-4) | Q99616 |
| Natural cytotoxicity triggering receptor 1 (NCR1) | O76036 |
| Natural killer cell receptor 2B4 (CD244) | Q9BZW8 |
| Natural killer cells antigen CD94 (KLRD1) | Q13241 |
| Nitric oxide synthase, endothelial (NOS3) | P29474 |
| Placenta growth factor (PGF) | P49763 |
| Platelet-derived growth factor subunit B (PDGF subunit B) | P01127 |
| Pleiotrophin (PTN) | P21246 |
| Pro-epidermal growth factor (EGF) | P01133 |
| Programmed cell death 1 ligand 1 (PD-L1) | Q9NZQ7 |
| Programmed cell death 1 ligand 2 (PD-L2) | Q9BQ51 |
| Programmed cell death protein 1 (PDCD1) | Q15116 |
| Stromal cell-derived factor 1 (CXCL12) | P48061 |
| T-cell surface glycoprotein CD4 (CD4) | P01730 |
| T-cell surface glycoprotein CD5 (CD5) | P06127 |
| T-cell surface glycoprotein CD8 alpha chain (CD8A) | P01732 |
| T-cell-specific surface glycoprotein CD28 (CD28) | P10747 |
| TNF-related apoptosis-inducing ligand (TRAIL) | P50591 |
| Tumor necrosis factor (Ligand) superfamily, member 12 (TWEAK) | O43508 |
| Tumor necrosis factor (TNF) | P01375 |
| Tumor necrosis factor ligand superfamily member 14 (TNFSF14 ) | O43557 |
| Tumor necrosis factor receptor superfamily member 12A (TNFRSF12A) | Q9NP84 |
| Tumor necrosis factor receptor superfamily member 21 (TNFRSF21) | O75509 |
| Tumor necrosis factor receptor superfamily member 4 (TNFRSF4 ) | P43489 |
| Tumor necrosis factor receptor superfamily member 9 (TNFRSF9) | Q07011 |
| Vascular endothelial growth factor A (VEGF-A) | P15692 |
| Vascular endothelial growth factor C (VEGFC) | P49767 |
| Vascular endothelial growth factor receptor 2 (VEGFR-2) | P35968 |

# **Supplemental Material #2**

Tables denoting the O-link measured proteins' values corresponding to the text figures denoting differences between demographic and clinical characteristics. Summary of statistically significant differences demonstrated as higher (red) or lower (blue) than an appropriate comparison group at standard ((p<0.05); pale shade) and more conservative (darker shade) The data are presented as mean + standard deviation. Description of abbreviations and corresponding UniProt numbers are listed in Supplemental Material #1

# **Supplemental Material #2 Figure 1A**

# **Supplemental Material #2 Figure 1B**

# **Supplemental Material #2 Figure 1C**

# **Supplemental Material #2 Figure 2A**

# **Supplemental Material #2 Figure 3A**

# **Supplemental Material #2 Figure 4A**

# **Supplemental Material #2 Figure 4B**

ECMO – extracorporeal membrane oxygenation.

# **Supplemental Material #2 Figure 5A**

CVf - cardiovascular failure.

# **Supplemental Material #2 Figure 5B**

CNSf - central nervous system failure.

# **Supplemental Material #2 Figure 5C**

Lf - liver failure.

# **Supplemental Material #2 Figure 6**

Rf - respiratory failure.

# **Supplemental Material #2 Figure 7**

AKIf - acute kidney injury failure.

# **Supplemental Pivot Table #1**

Pivotal table of measured markers and clinical correlates

# **Supplemental Figure #1**

Receiver operator curve for discharge to home demonstrated unsatisfactory values at the time of admission for CCL23, Gal-9, LAMP3, GZMB, LAG-3 (A) but not EGF (B).
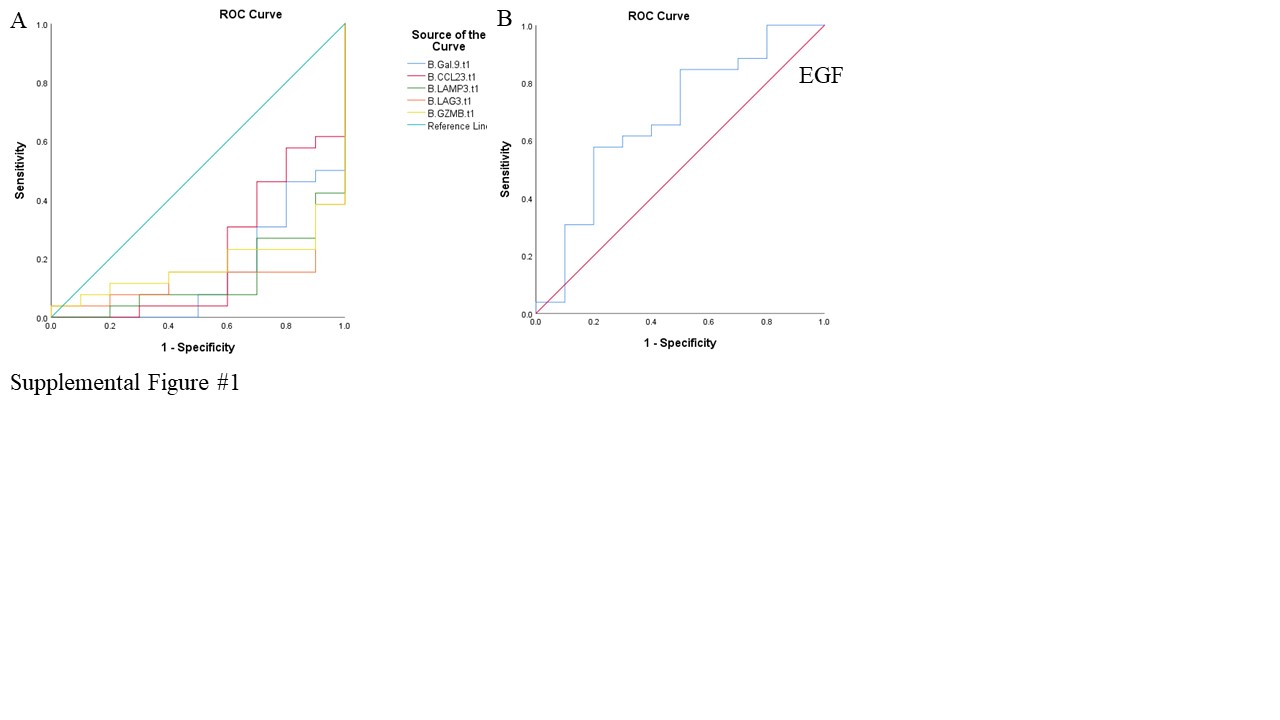


# **Supplemental Figure #2**

Most significant predictors of length of stay during regression modeling.


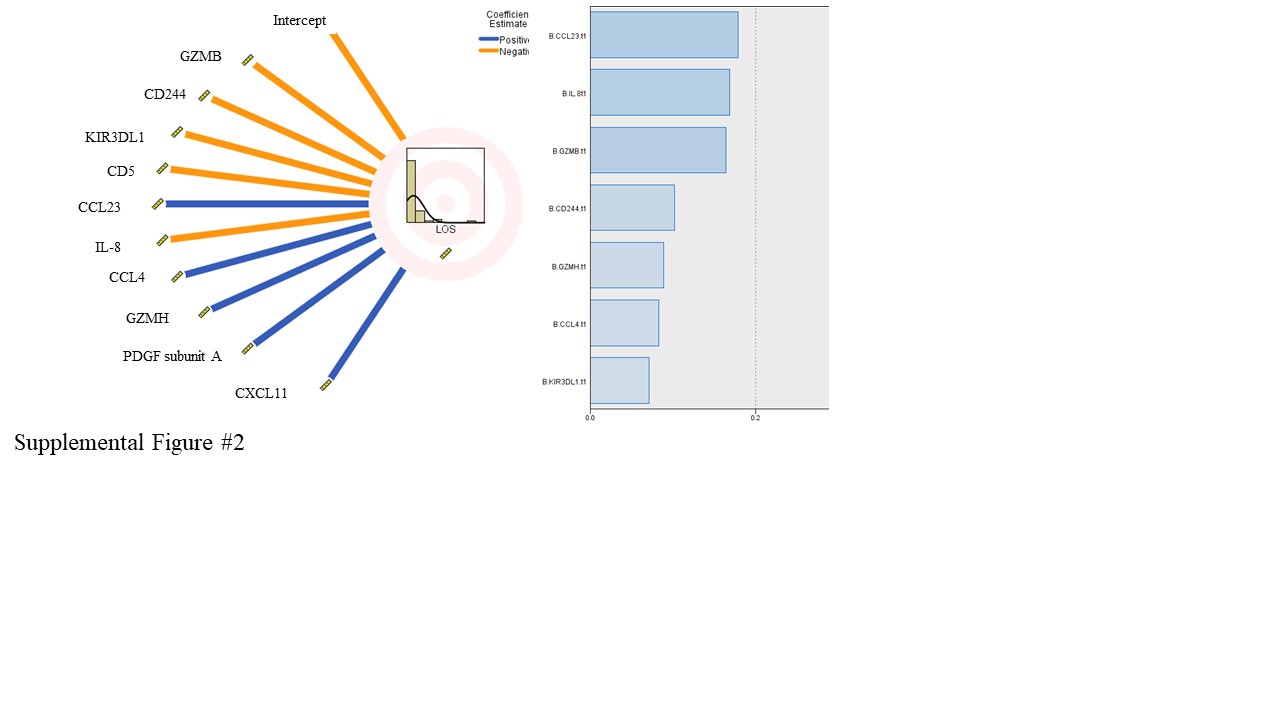


# **Supplemental Figure #3**

The occurrence of specific abnormalities in the serum levels of immunological markers during the natural history of COVID-19. The underlying color for biomarkers corresponds to the markers' organ failure frequency as compared to the subjects without organ failure in the given time frame (white – no changes; yellow – one system affected, orange – two systems affected, grey – three systems affected, red – four system affected, green – all organs affected). The blue square demonstrates a statistically significant difference at p<0.05 when a compared patient with organ failure to intact organ performance


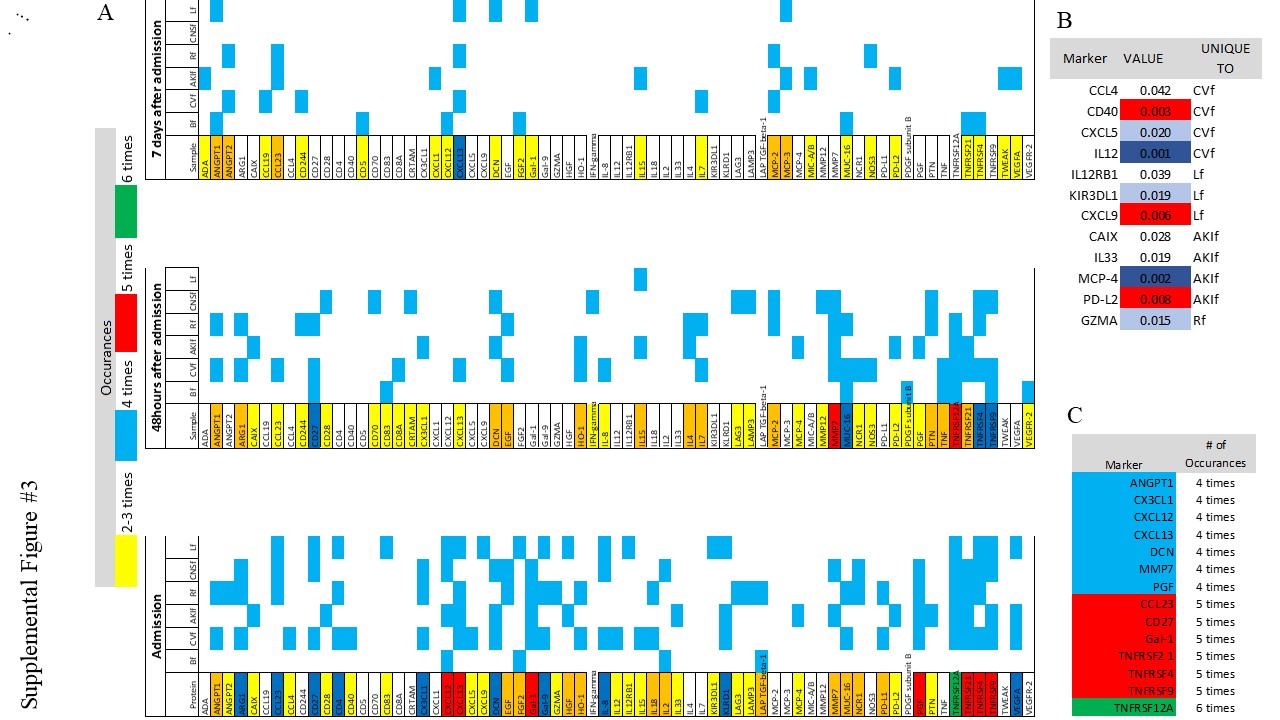


CVf - cardiovascular failure, Rf - respiratory failure, CNSf - central nervous system failure, AKIf - acute kidney injury failure, Lf - liver failure.

# **Supplemental Figure #4**

The effect of anti-COVID-19 treatment at different levels of molecular markers. Yellow fields denote significance at p<0.01 as compared to not treated patients.


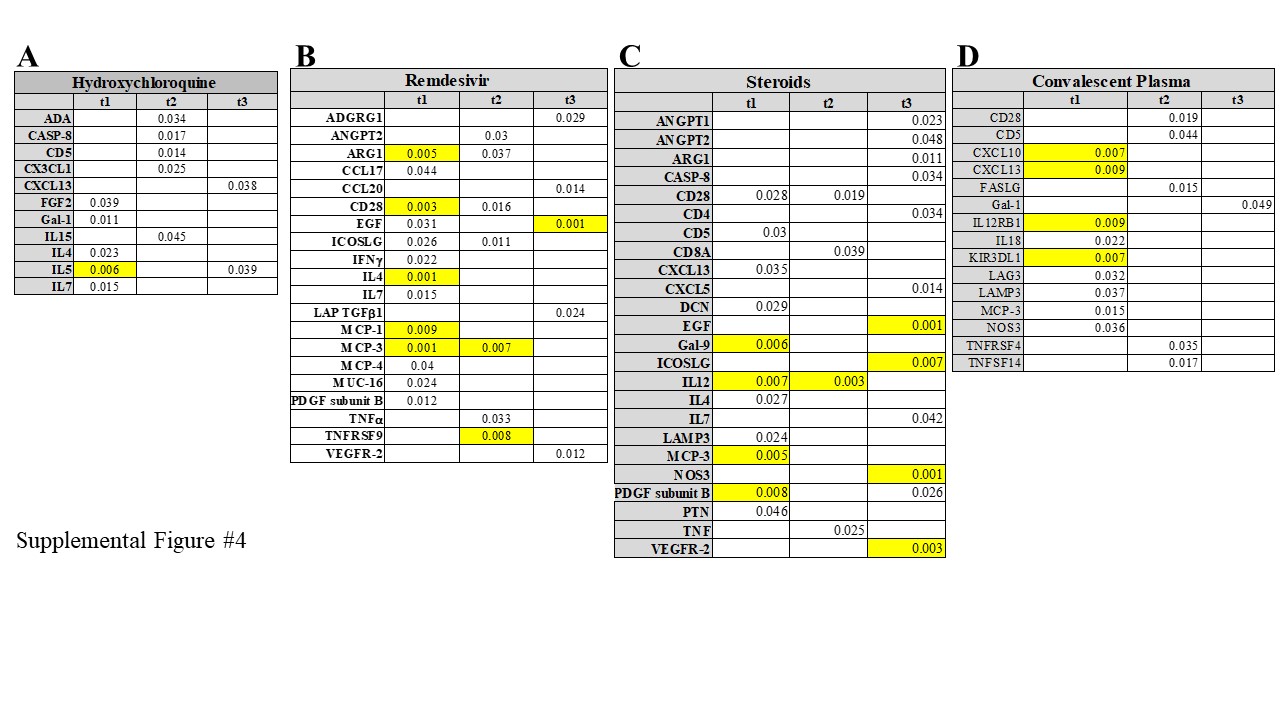

Supplement: Supplementary file 2 [file DataSheet_1.docx]
